# Novel Magnetic Zinc Oxide Nanotubes for Phenol Adsorption: Mechanism Modeling

**DOI:** 10.3390/ma10121355

**Published:** 2017-11-25

**Authors:** Marwa F. Elkady, Hassan Shokry Hassan, Wael A. Amer, Eslam Salama, Hamed Algarni, Essam Ramadan Shaaban

**Affiliations:** 1 Fabrication Technology Department, Advanced Technology and New Materials Researches Institute, City of Scientific Researches and technological applications, New Borg El-Arab City, Alexandria 21934, Egypt; 2 Chemical and Petrochemical Engineering Department, Egypt-Japan University of Science and Technology, New Borg El-Arab City, Alexandria 21934, Egypt; 3 Electronic Materials Researches Department, Advanced Technology and New Materials Researches Institute, City of Scientific Researches and technological applications, New Borg El-Arab City, Alexandria 21934, Egypt; 4 Chemistry Department, Faculty of Science, Tanta University, Tanta 31527, Egypt; wael.amer@science.tanta.edu.eg; 5 Environment and Natural Materials Research Institute (ENMRI), City of Scientific Research and Technological Applications, New Borg El-Arab City, Alexandria 21934, Egypt; 6 Research Center for Advanced Materials Science (RCAMS), King Khalid University, Abha 61413, P.O. Box 9004, Saudi Arabia; halgarni@hotmail.com; 7 Physics Department, Faculty of Science, King Khalid University, Abha 61413, P.O. Box 9004, Saudi Arabia; 8 Physics Department, Faculty of Science, Al-Azhar University, Assiut 71452, Egypt; esam_ramadan2008@yahoo.com

**Keywords:** magnetic nano-zinc oxide, nanotube structure, microwave technology, phenol uptake process

## Abstract

Considering the great impact of a material’s surface area on adsorption processes, hollow nanotube magnetic zinc oxide with a favorable surface area of 78.39 m^2^/g was fabricated with the assistance of microwave technology in the presence of poly vinyl alcohol (PVA) as a stabilizing agent followed by sonic precipitation of magnetite nano-particles. Scanning electron microscopy (SEM) and transmission electron microscopy (TEM) micrographs identified the nanotubes’ morphology in the synthesized material with an average aspect ratio of 3. X-ray diffraction (XRD) analysis verified the combination of magnetite material with the hexagonal wurtzite structure of ZnO in the prepared material. The immobilization of magnetite nanoparticles on to ZnO was confirmed using vibrating sample magnetometry (VSM). The sorption affinity of the synthesized magnetic ZnO nanotube for phenolic compounds from aqueous solutions was examined as a function of various processing factors. The degree of acidity of the phenolic solution has great influence on the phenol sorption process on to magnetic ZnO. The calculated value of ΔH^0^ designated the endothermic nature of the phenol uptake process on to the magnetic ZnO nanotubes. Mathematical modeling indicated a combination of physical and chemical adsorption mechanisms of phenolic compounds on to the fabricated magnetic ZnO nanotubes. The kinetic process correlated better with the second-order rate model compared to the first-order rate model. This result indicates the predominance of the chemical adsorption process of phenol on to magnetic ZnO nanotubes.

## 1. Introduction

Nanotechnology has become one of the most active research areas in modern materials science. Generally, it is well known that the intrinsic properties of nanomaterials are inversely proportional to their size. So, whenever the nano-size of materials decreases, their exclusive properties such as catalysts, photoactivity, adsorptive and their various motivating features and applications will be enhanced [1]. The application of nanotechnology in water and wastewater treatment processes provides an innovative treatment capability and guaranteed economic results [2]. The nanomaterials are characterized by their effectiveness and a high capacity for decontaminating pollutants from wastewater compared to their larger counterpart materials. These characteristic features adapt their performance at water and wastewater treatment applications. Phenolic pollutants are considered priority pollutants in the water stream since they are harmful to organisms at low concentrations, can be toxic when present at elevated levels, and are known or suspected to be carcinogens. They are discharged mainly into environmental water from many industries, such as petroleum-refining, high-temperature coal conversion, resins and plastics. As they are highly harmful compounds, there is a necessity for phenol decontamination in industrial wastewater to preserve an environment that is clean and safe. Nowadays, different treatment processes are used for the removal and/or recovery of phenols such as hot gas or steam-stripping [3], adsorption [4], ion exchange [5], plasma oxidation [6], electrochemical [7], photo-catalysis [8] and biological treatment processes [9]. Among the different phenol decontamination techniques, the adsorption method represents the most appropriate technique for the removal of aromatic pollutants from contaminated wastewater compared with traditional utilized techniques such as electrochemical oxidation [10], coagulation, flocculation [11], photochemical destruction [12] and membrane filtration [13,14]. The adsorption capabilities of many nanomaterials and their composites such as nano-zeolites [15,16,17,18], nano-ion exchange materials [19,20,21] and nano-metal oxides [22] have been investigated extensively. 

Recently, nano-metal oxides, especially ZnO, have received considerable attention in the field of water remediation due to their unique structures and properties, such as the wide band gap in the near-ultraviolet (UV) spectral region, strong oxidation ability, and good photocatalytic property. The ZnO nanostructure is an environment-friendly material, as it is non-toxic for different organisms [23], which makes the material suitable for the treatment of water and wastewater. Besides, the photocatalytic capability of the ZnO nanostructure is similar to that of the TiO_2_ nanostructure because their band gap energies are almost the same [24]. However, the ZnO nanostructure has the advantage of low cost alongside its biocompatibility compared with the TiO_2_ nanostructure. So, various ZnO morphological nanostructures have been prepared such as nanoparticles (NPs), nanorods, nanowires, nanosheets, nanodumbbells, nanobelts, nanotubes, nanotetrapods and nanoflowers using various techniques [25]. Sol-gel, hydrothermal methods, electrochemical depositions, thermal deposition and combustion methods have been utilized to produce ZnO in various forms. Recently, microwave-assisted synthesis technology has been widely used not only to help in processing mono dispersed and highly homogeneous nanomaterials; but also for controlling the morphology of nanomaterials. So, the synthesis of ultrafine ZnO nanostructures with controlled morphology using microwave irradiation technology has been carried out in recent research [26] in order to produce ZnO in a specific morphological structure. This technique is considered to be a novel route to fabricating ZnO in a nanotube structure [21]. The nanotube structure possesses several different areas of contact (borders, inner and outer surfaces, and structured tube walls) that in principle can be functionalized in several ways. Moreover, the tubular nanostructures might exhibit some interesting physical and chemical properties unattainable in other nanostructures and open up possibilities for various new applications [27]. In spite of the interstitial properties of nanostructured ZnO, the light absorption of ZnO nanomaterial as with TiO_2_ nanomaterial is limited due to their large band-gap energies [28]. Moreover, the handling of very fine ZnO nano-powdered material either in batch or continuous techniques in the water remediation process is very difficult [29]. In order to overcome all these limitations of ZnO nanomaterials, recently the synthesis of various nanocomposites, especially ferromagnetic nanocomposite materials, has become the most active subject in the field of nanomaterials.

One approach for producing ferromagnetic ZnO nanomaterials is to introduce small amounts of magnetic ions (Co, Ni, Fe or Mn) into the non-structured ZnO, creating what is called diluted magnetic semiconductors (DMS). ZnO-based DMS have attracted a great deal of research attention and controversy over the past decade [28]. Although there are many papers reporting on ZnO-based DMS nanomaterials and their related applications, the main goals of this work differ. The basic idea of this research is to add magnetism to ZnO nanostructure material, keeping as many intrinsic properties of ZnO as possible [30]. This idea is implemented through the functionalization of the ZnO surface, by coupling it with iron oxide (magnetite) NPs. There is no need to utilize surfactants or organic ligands trapped at the interface, as previously described for the production of Fe_3_O_4_/ZnO core-shell magnetic nanoparticles [31]. On the contrary, it is important to keep surface fractions free from the two coupled metal oxides (ZnO and Fe_3_O_4_) in the composite material as much as possible. These surface fractions of the coupled metal oxides will add new multifunctional properties. In recent research, the core-shell composite nanoparticles were utilized for the photo-degradation of organic dyes and phenols from polluted water [32]. However, the surface functionalization of ZnO nanostructure with iron oxide NPs has not been sufficiently investigated. Therefore, the aim of this work to focus mainly on the surface functionalization of prepared ZnO nanotubes with iron oxide (Fe_3_O_4_) NPs to produce magnetic ZnO composite nanotubes that can be utilized for phenol decontamination in polluted water. The physicochemical properties of prepared magnetic zinc oxide nanotubes were examined using various characterization techniques such as X-ray diffraction (XRD), scanning electron microscopy (SEM), transmission electron microscopy (TEM) and a vibrating sample magnetometer (VSM). The mechanism of the phenol sorption process on to synthetized magnetic zinc oxide nanotubes was investigated through equilibrium, kinetic and thermodynamic analysis of the experimental data. 

## 2. Materials and Methods 

### 2.1. Synthesis of Magnetic Zinc Oxide Nanotubes

Firstly, ZnO nanotube was prepared via microwave technique in presence of poly vinyl alcohol (PVA) as a stabilizing agent. Aqueous solution of zinc acetate (14 mM) Zn(CH_3_COO)_2_·2H_2_O (Rankem, Gurgaon, India) was prepared by dissolving 8 g of zinc acetate in 150 mL of distilled water. Then, 25 mg of PVA (Sigma-Aldrich, Darmstadt, Germany) was mixed with zinc salt as a stabilizing agent, and sodium hydroxide (NaOH) (Sigma-Aldrich, Darmstadt, Germany) was added for zinc salt reduction. The prepared aqueous solution was maintained in a microwave (THOMSON-COMBI1, Thomson Premier Lighting & Appliance, Logan, UT, USA) for 1 h at 800 W. Subsequently, for magnetite immobilization, 0.5 g of the prepared nano-zinc oxide was suspended using a direct sonication probe ultrasonic homogenizer (Vibra-Cell VCX 500, SONICS, Newtown, CT, USA) in 200 mL mixed solution of iron (III) chloride and iron (II) sulphate with a molar ratio of 2:1 until homogeneous suspension was obtained. A sodium hydroxide solution of 5 M was added dropwise to the previous suspension at 70 °C and maintained for 30 min under constant stirring until black precipitate of magnetic zinc oxide synthesised. The obtained black powders were washed several times with distilled water and absolute ethanol, and then separated using centrifugation at a force at 4000 rpm. Finally, the nanopowders were dried at 70 °C overnight. 

### 2.2. Characterization of Magnetic ZnO Nanotubes

X-ray patterns of both ZnO before and after magnetite immobilization were determined using a (Schimadzu-7000, Shimadzu Corporation, Kyoto, Japan) diffractometer that was operated by Cu Kα radiation (λ = 0.15406 nm) produced at 30 kV and 30 mA. The prepared nanopowder materials were packed into a flat aluminum holder. The scan velocity was fixed at 2°·min^−1^ from 10° to 80°.

The morphological structure of the prepared magnetic ZnO nanotubes was determined with scanning electron microscopy (JEOL JSM 6360LA, JEOL, Tokyo, Japan). The magnetic nanopowder material was stacked and gold sputtered over the aluminum holder to be examined. The scanning was carried out in order to recognize the morphological structure of the prepared material at different magnifications. The morphological structure of the synthesized composite was confirmed using a transmission electron microscope (TEM, JEM-2100, JEOL, Tokyo, Japan). The material’s specific surface area, BET (Brunauer-Emmett-Teller), was measured using a nitrogen adsorption analyzer (Beckman Coulter SA3100, Brea, CA, USA).

The magnetic properties of the prepared magnetic material were analyzed using a vibrating sample magnetometer (VSM, Dexing, Model: 250, Lake Zurich, IL, USA) at room temperature.

Finally, the point of zero charge (pH_pzc_) of the prepared magnetic ZnO material was determined through mixing 0.15 g from the material with 50 mL of 0.1 molar NaCl. The solution’s pH was adjusted so that it could be maintained within the range 1–12 using 0.01 M NaOH and/or 0.01 M HCl. Equilibration was realized by shaking in a thermostatic bath for 24 h at 25 °C. The dispersions were then separated and the final pH of the solutions was measured. The final solution pH was plotted against the initial pH. The pH value at which the plotted curve intersects the line of pH (final) = pH (initial) was taken as the pH_pzc_ of the prepared magnetic ZnO material surface.

### 2.3. Batch Investigation of Magnetic Zinc Oxide Nanotubes

The phenol adsorption capacity of the magnetic zinc oxide nanotubes was carried out by the batch contact method [24]. In batch method, 20 mg of nano-zinc oxide were added to 10 mL of known phenol solution concentrations and mixed at different temperatures using a shaking incubator. The phenolic solution’s pH was controlled with the addition of small quantities of HCl or NaOH (0.1 M). At the end of each batch test, the treated solution was centrifuged to determine the phenol concentration in the supernatant and measured using the colorimetry method with a UV spectrophotometer at 510 nm. The percentage removal of phenol using magnetic zinc oxide adsorbent was estimated from Equation (1):

% phenol removal = ((C_i_ − C)/C_i_) × 100 (1)

where C_i_ is the initial phenol concentration in solution (mg/L); and C is the final phenol concentration in aqueous solution after phase separation (mg/L). The phenol uptake capacity was determined by calculating the uptake amounts per gram of magnetic zinc oxide from the following Equation (2):

Q (mg/g) = V (C_i_ − C)/M (2)

where Q is the phenol adsorption capacity (mg/g); V is the phenol volume (mL); and M is the mass of magnetic zinc oxide nanotubes (g). The effect of the processing parameters’ variation on the performance of phenol sorption on to the synthesized magnetic ZnO nanotubes was screened over the following studied ranges: contact time (0–180 min), solution pH (1–11), material dose (1–20 g/L), initial phenol concentration (5–100 ppm) and solution temperature (25–85 °C). The accuracy and reliability of the collected data were confirmed by performing all adsorption experiments in triplicate, and the mean values were used in data analysis. The calculated relative standard deviations were found to be within ±2%.

### 2.4. Kinetics and Thermodynamics Studies

The thermodynamic parameters of the phenol adsorption process were evaluated to determine the nature of the process. The equilibrium behavior of the phenol sorption process on to magnetic ZnO nanotubes was examined using Langmiur, Frendlich and Temkin equilibrium isotherm equation models. The kinetics of the phenol sorption process were tested using the Pseduo-first-order, Pseduo-second-order and Elovich kinetic equation models.

## 3. Results and Discussion

### 3.1. Characterization of Hollow Structured Magnetic Zinc Oxide Nanotubes

**Figure 1:**
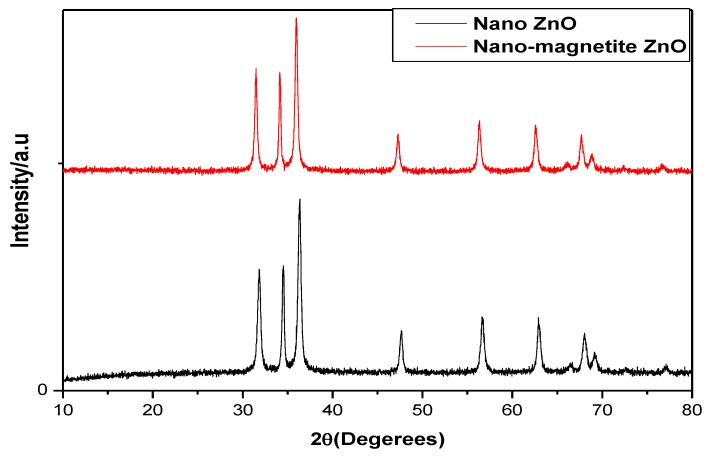
X-ray diffraction (XRD) patterns for pure ZnO nanotubes and magnetic ZnO nanotubes.

XRD patterns of both microwave-synthesized ZnO nanotubes and the magnetic composite material are investigated in Figure 1. The results indicate that both prepared materials have the same identical characteristic peaks. The main characteristic peaks of the hexagonal wurtzite ZnO structure are assigned at 2θ = 31.74°, 36.83° and 47.62°, which correspond to (100), (101) and (102) planes. On the other hand, the main characteristic peaks of the immobilized magnetite on to the ZnO surface are assigned at 2θ = 31.2°, 35.9°, 46.2°, 57.4°, 64.3°, which correspond to (220), (311), (400), (511) and (440) planes. So, the main characteristics peaks of ZnO at the magnetic ZnO match the main characteristic peaks of pure ZnO that show symmetry in XRD patterns of both pure ZnO and magnetic ZnO (Figure 1). The high-intensity degree of the peaks implies high materials crystallinity. It can be elucidated from Figure 1 that there are no characteristic peaks for impurities either in the pure ZnO or magnetic ZnO [19]. Accordingly, the prepared materials were pure materials without any contaminates from the PVA stabilizing agent.

**Figure 2:**
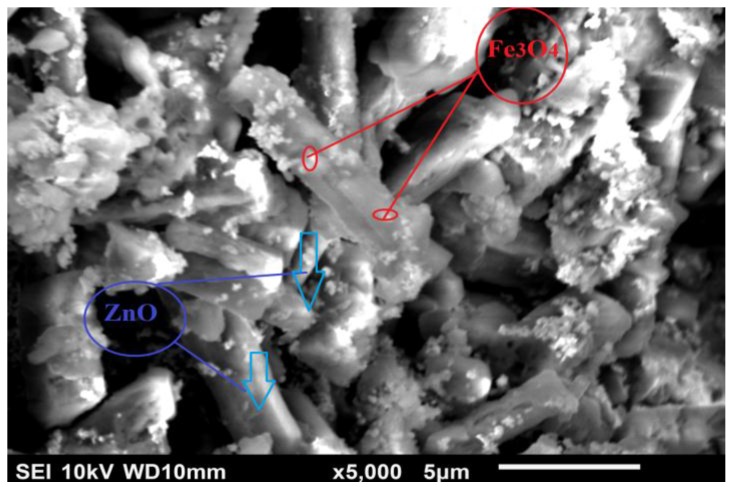
Scanning electron microscopy (SEM) images of the magnetic ZnO nanotubes.

The morphological structure of the synthesized magnetic zinc oxide is investigated in Figure 2. It is clear that, firstly, ZnO was prepared in a hollow nanotube structure. The nanotube formation may be due to the combination of microwave radiation in the presence of weak basic PVA surfactant material. The presence of PVA surfactant in the reaction media not only accelerates the reaction of the growth units, but also leads to their oriented growth by reducing the solution surface tension, which decreases the energy needed to form a new phase. The mechanism of ZnO nanotube formation in the microwave in the presence of PVA may be explained by the Kirkendall effect. In the Kirkendall effect, the diffusion of atoms causes oversaturation of lattice voids. It is considered that this oversaturation causes condensation of more voids close to the interface. Therefore, these Kirkendall voids change the properties of the interface and force it to form hollow nanotubes after ageing time of 1 h [33].

**Figure 3:**
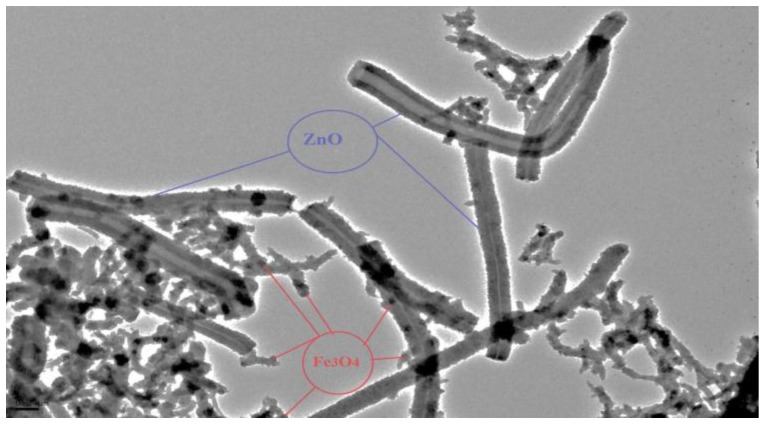
Transmission electron microscopy (TEM) images of the magnetic ZnO nanotubes.

TEM analysis was applied to study the detailed structural features of magnetic nano-zinc oxide at Figure 3. The TEM image of magnetic ZnO nanotubes reveals that the Fe_3_O_4_ nanoparticles are uniformly distributed on the plane of ZnO nanotube surfaces and the magnetite particles successfully wrap around the surface of the ZnO. 

**Figure 4:**
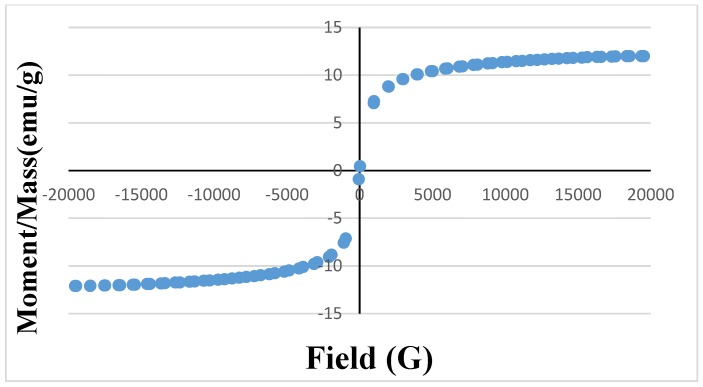
Magnetization curves of magnetic ZnO nanotubes.

The magnetic properties of the magnetic zinc oxide nanotubes have been investigated using a vibrating sample magnetometer at room temperature, as shown in Figure 4. The hysteresis loop of the synthesized magnetic zinc oxide nanotubes is recorded in Figure 4. It is clear that the material is a typical superparamagnetic. The saturation moment per unit mass, Ms, for the magnetic nano-zinc oxide is 12.0052 emu/g. These properties are assigned to the immobilized magnetite nanoparticles on to zinc oxide nanotubes. The results agree with a previous study, that magnetite nanoparticles exhibit superparamagnetic properties when they are smaller than the critical size of the magnetic domain size [34].

### 3.2. Magnetic Zinc Oxide Nanotubes for Phenol Removal

The feasibility of the synthesized magnetic zinc oxide nanotubes for phenol sorption from synthetic wastewater was examined.

#### 3.2.1. Influence of Contact Time on the Phenol Sorption Process on to Magnetic ZnO Nanotubes

**Figure 5:**
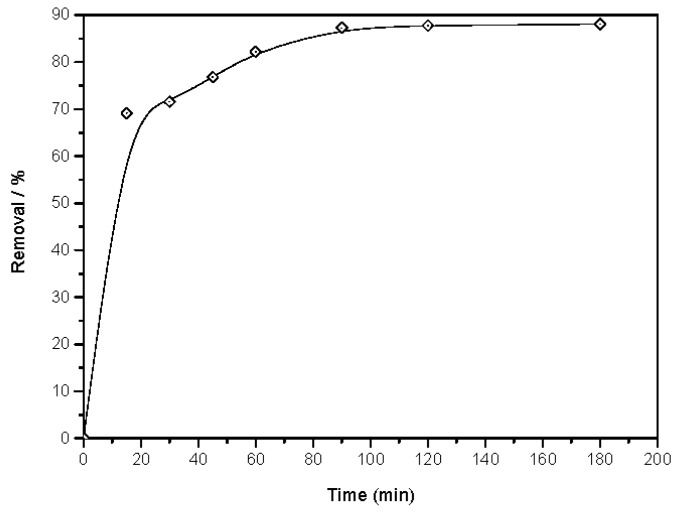
Influence of contact time on phenol sorption process on to magnetic ZnO nanotubes (pH = 5, initial phenol concentration = 10 ppm, agitation speed = 440 rpm, material dosage = 2 g/L and temperature = 25 °C).

The influence of contact time on the adsorption of the phenol on to magnetic zinc oxide nanotubes was investigated at up to 180 min time intervals. From Figure 5, it is clear that in the initial period, the phenol adsorption on to the magnetic material is a rapid process that then decreases with time until reaching the equilibrium state. The fast adsorption of phenol relates to the high available surface area of the prepared magnetic ZnO nanotubes that equal 51.45 m^2^/g. The quantity of phenol ions adsorbed on to the magnetic nanomaterial is at dynamic equilibrium with phenol ions desorbed from the material [35]. The equilibrium time of the phenol sorption process is recorded within 90 min, with maximum phenol percentage removal of 87.75% under these particular conditions.

#### 3.2.2. Influence of Magnetic Nano-ZnO Dosage on the Phenol Adsorption Process

**Figure 6:**
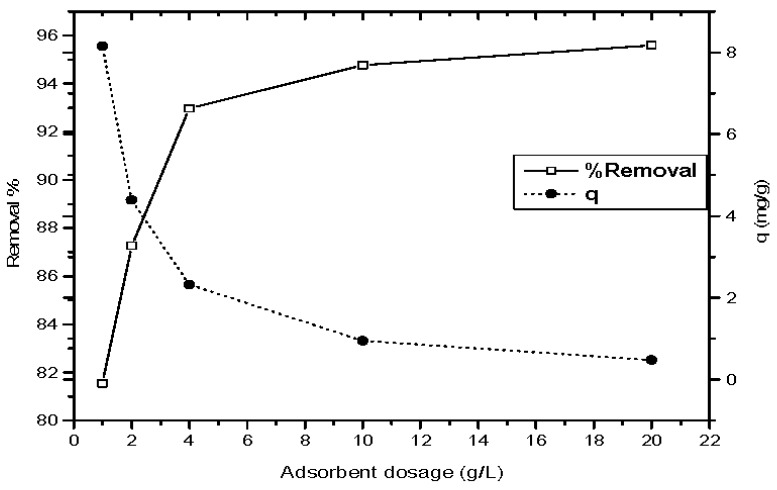
Influence of magnetic ZnO nanotubes dosage on both phenol percentage removal and phenol uptake capacity (pH = 5, initial phenol concentration = 10 ppm, agitation speed = 440 rpm, contact time = 90 min and temperature = 25 °C).

Adsorbent dosage represents an important parameter due to its strong effect on the capacity of an adsorbent at a given initial concentration of the adsorbate [36]. The influence of magnetic ZnO nanotube dosage on both percentage phenol removal and material sorption capacity was traced after 90 min. Figure 6 reveals that the phenol removal percentage is enhanced from 81.54 to 95.59% as magnetic ZnO nanotubes dosages improved from 1 to 20 g/L, and a decrease of the particles sorption capacity is noticed with increasing material dosage. The decline at unit adsorption with increasing particle concentration may be due to the remaining of unsaturated adsorption sites on the magnetic ZnO adsorbent material [37]. However, the increase in the magnetic ZnO nanotube dosage increases the availability of more active sites available for the phenol adsorption process. These results may be owing to the large determined active surface area of the prepared hollow-structured magnetic ZnO which is the equivalent 51.45 m^2^/g. 

It obvious from Figure 6 that the optimum ZnO dosage is recorded as 2.5 g/L at the line’s intersection between the phenol percentage removal and material adsorption capacity. However, the ZnO dosage of 2 g/L studied represents the closed economical dosage to the recorded value of the lines’ intersection. So, the optimum magnetic ZnO dosage that achieved the optimum phenol removal percentage of 87.75% and adsorption capacity of 3 mg/g is considered to be 2 g/L.

#### 3.2.3. Influence of Initial pH on the Phenol Adsorption Process

**Figure 7:**
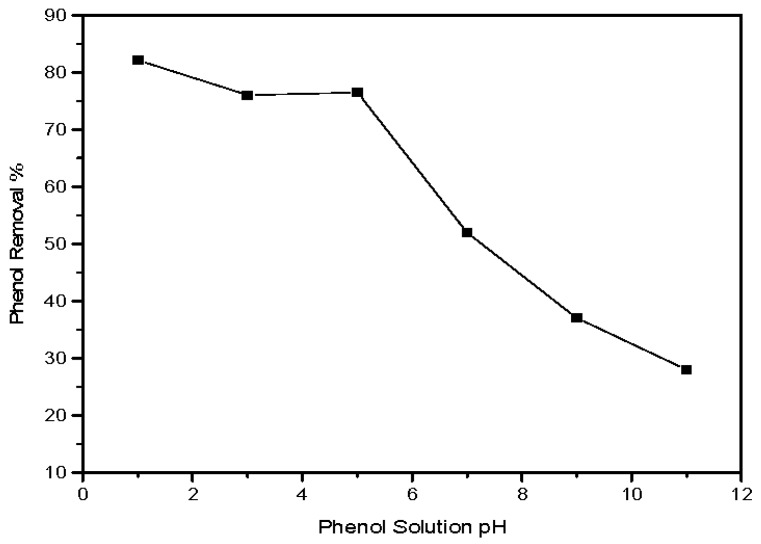
Influence of solution pH on the percentage of phenol removal on to magnetic ZnO nanotubes (initial phenol concentration = 10 ppm, material dosage = 2 g/L, agitation speed = 440 rpm, contact time = 90 min and temperature = 25 °C).

The solution’s pH has a significant impact on control of the phenol adsorption process on to magnetic ZnO nanotubes, affecting the surface charge of the adsorbent material as well as the degree of ionization of phenol considerably [36]. It can be observed from Figure 7, that the adsorption is high at lower pH, meaning the magnetic ZnO has greater adsorption capacity in an acidic medium. The adsorption percentage of phenol is high at pH = 5 and gradually decreases with increasing pH of the solutions; the lowest adsorption is observed at pH = 11. The vital factor for phenol adsorption on to the prepared material is the pH_pzc_ of magnetic ZnO. The point zero charge (pzc) of the prepared magnetic ZnO was recorded experimentally to be around 6 (figure not included). So, at any pH below the pzc, the surface of the magnetic ZnO material is positively charged; and above the pzc, it is negatively charged. The optimum adsorption of phenol on to the magnetic material was recorded at pH = 5. This result can be attributed to both the phenol ionization capacity and point zero charge (pH_pzc_) of magnetic ZnO. As the pH of the solution increases, the percentage of unionized species of phenol decreases and the ionized species increases. This is due to the fact that the pKa value of phenol is ~9.8 [37]. At solution pH = 5 (pH 5 < pH_pzc_), the magnetic ZnO surface is positively charged, so there is no electrostatic repulsion between the unionized phenol species and the positively charged surface that increases the adsorption process. However, as the solution pH increases (pH 7 > pH_pzc_), the adsorbent surface is negatively charged, reducing phenol adsorption due to the repulsive force between the phenolate ion and the negative charge of the magnetic ZnO surface [38].

#### 3.2.4. Influence of Initial Phenol Concentration on the Adsorption Process

**Figure 8:**
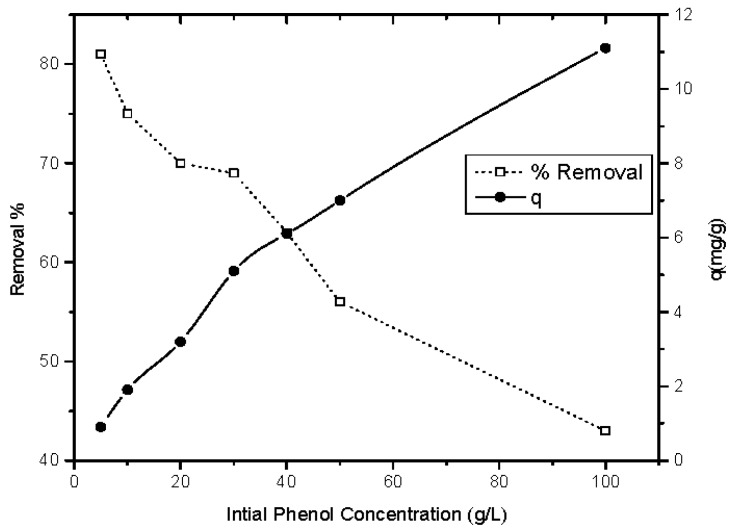
Influence of initial phenol concentration on both the percentage of phenol removal and phenol uptake capacity on to magnetic ZnO nanotubes (pH = 5, material dosage = 2 g/L, agitation speed = 440 rpm, contact time = 90 min and temperature = 25 °C).

The influence of initial phenol concentration on the phenol sorption capacity (q) at equilibrium was carried out in the range of 5–100 mg/L at a pH value of 5 using 2 g/L of magnetic ZnO nanotubes for 90 min. Figure 8 shows that there is an enhancement in the phenol sorption capacity as the initial concentration of the phenol increases from 5–100 mg/L, which is in accordance with other reported research [30]. This behavior may be attributed to the saturation of the adsorption sites on the magnetic ZnO nanotubes as the concentration of the phenol increases. According to these results, the fabricated magnetic zinc oxide nanotubes are effectively capable of removing the phenol from solutions with a variable initial concentration range completely.

#### 3.2.5. Influence of Solution Temperature on the Phenol Adsorption Process

**Figure 9:**
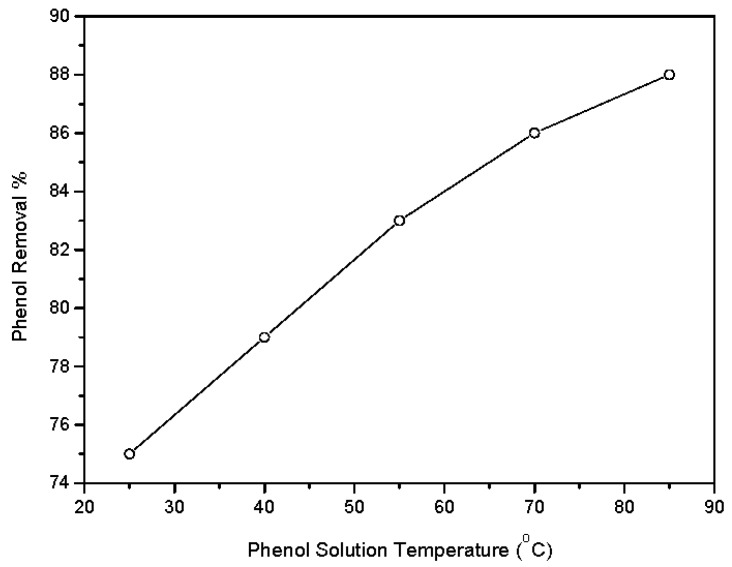
Influence of phenol solution temperature on the percentage of phenol removal on to magnetic ZnO nanotubes (initial phenol concentration = 10 ppm, material dosage = 4 g/L, agitation speed = 440 rpm, contact time = 90 min and pH = 5).

Figure 9 elucidates the impact of the phenol solution’s temperature (25–85 °C) on the percentage of phenol removal on to the synthesized magnetic ZnO nanopowder. Moreover, this figure indicates that rising temperature enhances the phenol decontamination process. This behavior may be owing to the chemical interaction between phenol and magnetic ZnO material. Moreover, the rate of intraparticle diffusion of phenols into the pores of the magnetic adsorbent is enhanced, and that may create new reaction sites on to the magnetic ZnO at high solution temperature [39]. These results indicate that phenol sorption is an endothermic process. 

### 3.3. Thermodynamics and Equilbrium Modeling

Temperature dependence of the phenol adsorption process on to hollow-structured magnetic ZnO nanotubes is associated with various thermodynamic parameters, where the standard Gibbs free energy of the process is evaluated from Equation (3) [38]:

(3) $$\Delta G^{0}=-RTlnk_{c}$$

The equilibrium constant k_c_ is estimated at each phenol solution temperature as indicated in Equation (4):

(4) $$k_{c}=\frac{C_{Be}}{C_{Ae}}$$

where C_Be_ and C_Ae_ represent the equilibrium concentrations of phenol on magnetic ZnO adsorbent and solution, respectively. Standard enthalpy (ΔH^0^) and entropy (ΔS^0^) are calculated from the Van’t Hoff Equation (5):

(5) $$lnk_{c}=\frac{\Delta S^{0}}{R}-\frac{\Delta H^{0}}{RT}$$

Activation Energy (E_a_) is determined from the solution reaction relationship between E_a_ and ΔH^0^ [24]:

E_a_ = ΔH^0^ + RT (6)

ΔH^0^ and ΔS^0^ are obtained from the slope and intercept of the plot of lnK_c_ against 1000/T and the data are tabulated in Table 1. The calculated values of free energy changes ΔG^0^ have negative charges that allow prediction about the spontaneous and thermodynamically favorable nature of the phenol adsorption process on to magnetic ZnO. The negative values of the ΔG^0^ depression with the rise in temperature increase the driving force of the adsorption process. Therefore, the enhancement in temperature has a positive impact on the sorption process of phenol. The positive value of ΔH^0^ designates that the adsorption process is endothermic in nature. The positive value of entropy change (ΔS^0^) shows an increase in disorder at the solid/liquid interface through the phenol sorption process [37].

#### 3.3.1. Equilibrium Isotherm Analysis for the Phenol Sorption Process on to Magnetic ZnO Nanotubes

The plot of C_e_/q_e_ versus C_e_ (figure not investigated) shows a straight-line trend with a high correlation coefficient fraction (R^2^ = 0.966) [39]:

(7) $$\frac{C_{e}}{q_{e}}=\frac{1}{q_{m}K}+\frac{C_{e}}{q_{m}}$$

where C_e_ is the equilibrium concentration of the adsorbate ions (mg/L); q_e_ is the amount adsorbed (mg/g); and q_m_ and K are Langmuir constants correlated to the maximum monolayer adsorption capacity (monolayer capacity) (mg/g) and energy of adsorption (L/mg), respectively. Accordingly, the Langmuir isotherm model may be adequate for designating the phenol sorption process on to the synthesized magnetic ZnO nanotubes. The Langmuir constants q_m_ and K are determined from the slope and intercept of the linear plot, respectively (Table 2).

The value of the correlation coefficient (R^2^ = 0.966) has good linearity. The dimensionless constant separation factor R_L_ representing the important characteristics of the Langmuir isotherm can be expressed in Equation (8):

R_L_ = 1/(1 + KC_0_) (8)

where C_0_ (mg/L) is the adsorbate initial concentration; and K (L/mg) is Langmuir constant. R_L_ indicates the shape of the isotherm which is unfavorable (R_L_ > 1), linear (R_L_ = 1), favorable (0 < R_L_ < 1), or irreversible (R_L_ = 0). The R_L_ value for the adsorption of phenol on to magnetic ZnO nanotubes is observed to be in the range 0 to 1; this range investigates the favorable adsorption method. These results can be explained, as the hypothesis of the Langmuir isotherm equation is based upon the monolayer coverage of adsorbate molecule over a homogeneous surface of the adsorbent material, which means that the material surface consists of similar active sites. These sites are similarly available for adsorption with equivalent adsorption energies. Consequently, a saturation point is recorded at equilibrium and no further adsorption will take place after this point. The adsorption process is supposed to take place at specific homogeneous sites on magnetic ZnO nanotubes and, once a phenol molecule occupies the active sites, the adsorption process finishes, allowing the prediction that the process may be chemisorption [38].

Additionally, the equilibrium sorption data were analyzed using the Freundlich linear form Equation (9), by plotting log q_e_ against log C_e_. The estimated constants (K_F_, n_F_) of Freundlich and the correlation coefficient R^2^ are tabulated in Table 2.

Log q_e_ = log K_F_ + 1/n_F_ log C_e_ (9)

where K_F_ and n_F_ are Freundlich constants correlated to the capacity and intensity of adsorption, respectively. The high correlation coefficient (R^2^ = 0.972) represents the agreement of the Freundlich equation with the experimental results of phenol sorption on to magnetic ZnO nanotubes. The best fit of the equilibrium data with the Freundlich isotherm assumes a heterogeneous adsorption surface with a non-uniform distribution of adsorption heat. The calculated n_F_ value of 1.63 is greater than unity, showing that the phenol sorption process on to magnetic ZnO nanotubes was favorable. As the phenol sorption process obeys the Freundlich model, this supposes that the multi-layer sorption of phenol on to magnetic ZnO nanotubes allows predictions about physical phenol adsorption on to the prepared material [36]. 

The adsorption data for phenol on magnetic ZnO nanotubes were analyzed to fit the Temkin isotherm model expressed as [39]:

q_e_ = B ln A + B ln C_e_ (10)

where, B = RT/b is constant correlates to sorption heat (J/mol); and A is the Temkin isotherm constant (L/g).

The Temkin model constants and the correlation coefficient are illustrated in Table 2. Comparing the correlation coefficient of the Temkin model with those for Langmuir and Freundlich, it obvious that the equilibrium data of the phenol sorption process on to magnetic ZnO follows both the latter models to a great extent.

#### 3.3.2. Kinetic Model of Phenol Adsorption Process on to Hollow Magnetic ZnO Nanotubes

It is necessary to ascertain the removal rate of dissolved phenol from aqueous solution using solid sorbent material in order to assess the kinetics of the adsorption process by theoretical modeling so as to control the adsorption process. This is so that the suitability of the pseudo-first-order, pseudo-second-order and Elovich models can be verified for phenol adsorption on to magnetic ZnO nanotubes. The best-fit of the kinetics model is designated based on the linear correlation coefficient values. The Lagergren first-order equation [40] is expressed as:

ln (q_e_ − q_t_) = ln q_e_ − k_1_t (11)

where, q_e_ and q_t_ are the amounts of phenol ions sorbed (mg/g) at equilibrium and at time (min), respectively; and k_1_ (min) is the first-order reaction rate constant. The calculated values of k_1_, q_e_ from the equation and the correlation coefficient (R^2^) value for fitting the first-order rate model are tabulated in Table 3. The linearity of ln (q_e_ − q_t_) plotting against time indicates that the phenol sorption process did not follow the first-order rate kinetic model [29]. Additionally, the calculated value of (q_ecal_) obtained from the equation differs significantly from the experimentally (q_eexp_) measured values. This confirms that the alkaline phenol sorption process using magnetic ZnO nanotubes did not follow the pseudo-first-order model.

Furthermore, the phenol sorption kinetic data are examined using the pseudo second-order kinetic model that may be represented as:

t/q_t_ = (1/k_2_q^2^) + t/q (12)

where k_2_ is the second-order reaction rate equilibrium constant (g/mg·min). The plot of t/q_t_ against time (figure not presented) shows a straight line with high correlation coefficient values, R^2^ (>0.99), compared with the first-order rate model (Table 3). Furthermore, the calculated value of q_e_ is very close to that obtained experimentally. Consequently, the second order kinetic model is more appropriate for describing the phenol sorption process on to the magnetic zinc oxide nanotubes that supposes a chemisorption process [41].

An Elovich equation is established for the multilayer adsorption, assuming that the adsorption sites increase exponentially with adsorption that may be written as:

q_t_ = α + β ln t (13)

where α signifies the initial sorption rate (mg/g·min) and β is correlated to the surface coverage degree and chemisorption activation energy (g/mg). The constants α and β can be calculated from the slope and intercept of the linear plot of q_t_ versus ln t and is tabulated in Table 3. This indicates that the correlation coefficient of Elovich fitting is equal to 0.878, suggesting that the sorption process cannot be explained by the Elovich model [38].

### 3.4. Phenol Desorption Process

The regeneration of the adsorbent is one of the most important factors, since it affects the overall cost of the adsorption process. Various attempts have been made previously to desorb phenol from different adsorbent materials using many elution reagents, such as hydrochloric acid, ethanol and sodium hydroxide. From all of these attempts, sodium hydroxide has been found to be the most favorable reagent for phenol desorption [42]. Consequently, phenol adsorbed on the magnetic ZnO nanotubes is desorbed using a NaOH solution at various strengths. Table 4 shows that the amount of phenol desorbed increases with increasing strength of NaOH solution. The results indicate that the solution strength of 0.2 N NaOH is sufficient for quantitative desorption of a 68% phenol fraction from the magnetic ZnO nanotubes. The elution of phenol from the magnetic ZnO nanotubes in the presence of 0.2 N NaOH may be attributed to the formation of a sodium salt of phenol which may facilitate desorption of phenol from the magnetic material surfaces [43]. Rengaraj et al. [44] found that approximately 0.14 N NaOH is required for quantitative desorption of phenol from activated carbon.

## 4. Conclusions

Hollow structured magnetic zinc oxide nanotubes were successfully synthesized using the microwave method in the presence of PVA as a stabilizing agent followed by sonic precipitation of magnetite nano-particles. The prepared material was examined using X-ray diffraction, scanning electron microscopy, transmission electron microscopy and vibrating sample magnetometer techniques to confirm its purity, nano-size and magnetic properties, respectively. Improvement in both the phenol solution temperature and material dosage has a positive impact on the percentage of phenol decontamination. Estimation of the thermodynamic parameters (ΔG^0^, ΔH^0^, ΔS^0^ and E_a_) demonstrates the spontaneous and endothermic nature of the phenol sorption process. However, a positive value of ΔS^0^ indicates increasing randomness at the solid/liquid interface during the adsorption of phenol on to magnetite ZnO nanotubes. On the other hand, the mathematical description of the phenol adsorption equilibrium on to magnetic zinc oxide nanotubes was tested using Langmuir, Freundlich and Temkin isotherm equations. This mathematical equilibrium modeling suggested that phenol decontamination using magnetite ZnO nanotubes includes both chemical and physical adsorption processes. Finally, three kinetics models comprising pseudo-first-order reaction rate, pseudo-second-order reaction rate and Elovich models were used to examine the phenol adsorption process. It was evident that the adsorption of phenol on to the magnetic zinc oxide nanotubes was described well by the pseudo-second-order reaction rate model, which allows a prediction that the studied sorption system is controlled by a chemisorption process. 

## Figures and Tables

**Table 1:** Thermodynamic parameters for phenol sorption on to magnetic ZnO nanotubes.

Temp.(K)	1000/T	C_Be_	C_Ae_	K_c_	lnK_c_	ΔG^0^ (kJ·mol^−1^)	E^0^ (kJ·mol^−1^)	ΔH^0^ (kJ·mol^−1^)	ΔS^0^ (J·mol^−1^·K^−1^)
298	3.356	8.775	1.225	7.163	1.969	−4.878	2.501	23.495	96.027
313	3.195	9.134	0.866	10.547	2.356	−6.119	2.626		
328	3.049	9.642	0.358	26.933	3.293	−8.983	2.750		
343	2.915	9.678	0.322	30.056	3.403	−9.706	2.875		
358	2.793	9.709	0.291	33.364	3.508	−10.44	3.000		

**Table 2:** Isotherm parameters of the three models, Langmuir, Freundlich and Temkin, for phenol removal on to magnetic ZnO nanotubes.

Isotherms	Parameters	Value
Langmuir	q_m_ (mg/g) k (L/mg) R^2^	20.408 0.107 0.966
Freundlich	K_F_ (mg/g)(L/mg)^1/n^ 1/n_F_ R^2^	1.122 0.612 0.978
Temkin	A (L/g) B (J/mol) R^2^	2.722 3.164 0.923

**Table 3:** Pseudo first-order, second-order and Elovich kinetic parameters for phenol removal on to magnetic ZnO nanotubes.

Kinetic Model	Parameter	Value
Pseudo-first-order	q_exp_ (mg/g)	4.388
	q_theor_ (mg/g)	1.817
	K_1_ (min^−1^)	0.033
	R^2^	0.872
Pseudo-second-order	q_exp_ (mg/g)	4.388
	q_theor_ (mg/g)	4.405
	K_2_ (g/mg·min)	0.042
	R^2^	0.994
Elovich kinetic model	q_exp_ (mg/g)	4.388
	q_theor_ (mg/g)	4.196
	α (mg/g·min)	2.158
	β (g/mg)	0.453
	R^2^	0.878

**Table 4:** Fraction of desorbed phenol from magnetic ZnO nanotubes using NaOH solution after being saturated by 10 ppm initial phenol solution for 90 min contact time (desorption contact time = 24 h).

Sodium Hydroxide Concentration	Percentage Phenol Desorbed
0.05 N	24.2
0.1 N	30.7
0.15 N	47.8
0.2 N	68
0.25 N	70
0.3 N	73.55
